# Accuracy of 3D Corrective Osteotomy for Pediatric Malunited Both-Bone Forearm Fractures

**DOI:** 10.3390/children10010021

**Published:** 2022-12-23

**Authors:** Kasper Roth, Eline van Es, Gerald Kraan, Denise Eygendaal, Joost Colaris, Filip Stockmans

**Affiliations:** 1 Department of Orthopedics and Sports Medicine, Erasmus University Medical Centre, Dr. Molewaterplein 40, 3015 GD Rotterdam, The Netherlands; 2 Department of Orthopedics, Reinier HAGA Orthopedic Centre, Toneellaan 2, 2725 NA Zoetermeer, The Netherlands; 3 Department of Development and Regeneration, Faculty of Medicine, University of Leuven Campus Kortrijk, Etienne Sabbelaan 53, 8500 Kortrijk, Belgium

**Keywords:** corrective osteotomy, three-dimensional, malunion, fracture, forearm, radius, pediatric

## Abstract

Re-displacement of a pediatric diaphyseal forearm fracture can lead to a malunion with symptomatic impairment in forearm rotation, which may require a corrective osteotomy. Corrective osteotomy with two-dimensional (2D) radiographic planning for malunited pediatric forearm fractures can be a complex procedure due to multiplanar deformities. Three-dimensional (3D) corrective osteotomy can aid the surgeon in planning and obtaining a more accurate correction and better forearm rotation. This prospective study aimed to assess the accuracy of correction after 3D corrective osteotomy for pediatric forearm malunion and if anatomic correction influences the functional outcome. Our primary outcome measures were the residual maximum deformity angle (MDA) and malrotation after 3D corrective osteotomy. Post-operative MDA > 5° or residual malrotation > 15° were defined as non-anatomic corrections. Our secondary outcome measure was the gain in pro-supination. Between 2016–2018, fifteen patients underwent 3D corrective osteotomies for pediatric malunited diaphyseal both-bone fractures. Three-dimensional corrective osteotomies provided anatomic correction in 10 out of 15 patients. Anatomic corrections resulted in a greater gain in pro-supination than non-anatomic corrections: 70° versus 46° (*p* = 0.04, ANOVA). Residual malrotation of the radius was associated with inferior gain in pro-supination (*p* = 0.03, multi-variate linear regression). Three-dimensional corrective osteotomy for pediatric forearm malunion reliably provided an accurate correction, which led to a close-to-normal forearm rotation. Non-anatomic correction, especially residual malrotation of the radius, leads to inferior functional outcomes.

## 1. Introduction

In midshaft forearm fractures, growth will not remodel angular deformity as it does in distal fractures [1]. Impairment in forearm rotation is a critical problem associated with malunions of the forearm bones [2]. Malunited diaphyseal forearm fractures in children leading to a severe restriction in pro-supination may require corrective osteotomies [3]. A conventional corrective osteotomy can be technically demanding due to the multiplanar deformity of both forearm bones [4]. In a series by Miyake et al., one patient even had a rotational malunion of the radius of 136°, which is difficult to assess precisely using two-dimensional (2D) radiographic planning. Recent advancements in three-dimensional (3D) planning and 3D printing of patient-specific instruments (PSIs) can aid the surgeon in achieving a more accurate correction. Non-anatomic correction of the bony anatomy in malunions, especially of the upper extremity, may lead to inferior functional outcomes. Several authors have stated anatomically accurate correction during 3D corrective osteotomy is highly desirable to achieve a good outcome [5,6]. Few studies have tested this assumption nor have examined the effectiveness of 3D corrective osteotomy for pediatric malunited forearm fractures concerning the radiographic accuracy of the correction [3,7]. This prospective study aimed to assess the accuracy of correction after 3D corrective osteotomy for pediatric forearm malunion and if anatomic correction influences the functional outcome.

## 2. Materials and Methods

**Figure 1:**
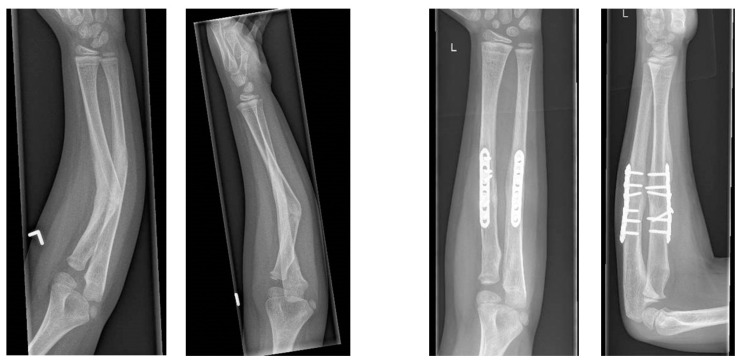
Example of pre- and post-operative radiographs.

This study represents an additional analysis of the radiographic outcomes of a prospective cohort of patients whose clinical outcomes have been published previously [8]. Patients were eligible for enrollment if they met the following inclusion criteria: having a symptomatic forearm malunion after a diaphyseal both-bone forearm fracture sustained during childhood (<18 years), resulting in a limitation in pro-supination (pronation or supination of <50°), with unsatisfactory improvement after physiotherapy and a minimum age of 10 years at 3D corrective osteotomy. In addition, patients were excluded if they had an osseous deformity of the contralateral forearm. The pre-operative planning, surgical technique, and post-operative management of our 3D corrective osteotomies are described in our previous publication [8]. Planning of 3D corrective osteotomy and 3D printing of PSIs were performed at Materialise N.V., Leuven, Belgium in collaboration with our surgeons. An example of pre- and post-operative radiographs is provided in Figure 1.

### 2.1. Outcome Measures

**Figure 2:**
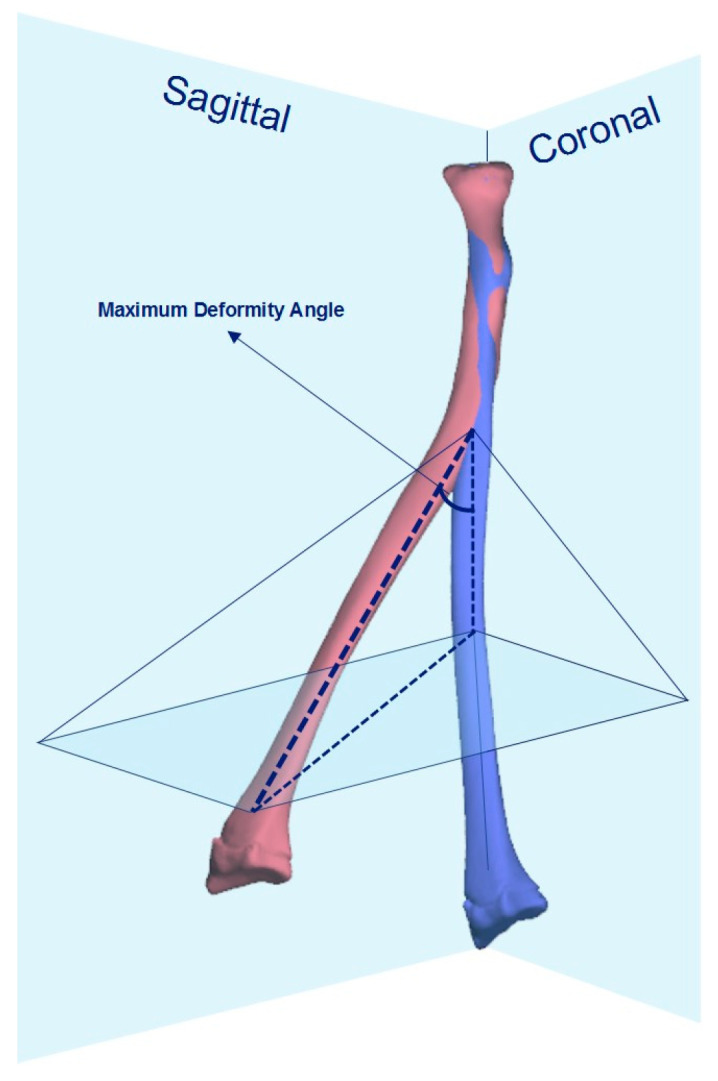
The maximal deformity angle (MDA) was calculated by combining the measurements of angular deformity in the coronal and sagittal plane according to the following formula:
$$MDA=\sqrt{tan^{2}\left(Coronal\right)+tan^{2}\left(Sagittal\right)\;}$$.

Our primary outcome measure was the radiographic accuracy of the achieved correction after 3D corrective osteotomy. To assess the accuracy of correction, we compared the 3D pre-operative plan with the one-year post-operative computed tomography (CT). The residual maximum deformity angle (MDA) and malrotation after 3D corrective osteotomy were used to describe the accuracy of correction. The MDA is calculated by combining the angular deformity on both the coronal and sagittal plane derived from CT, as described by Nagy et al., illustrated in Figure 2 [3,9]. Similar to the study by Byrne et al., we assessed how often angular deformities could be corrected to within 5° of contralateral by 3D corrective osteotomy. Residual MDA ≥ 5° was defined as a non-anatomic correction. Unlike for the lower extremity, which most authors recommend to correct a torsional deformity of ≥15° [10], there are still no uniform cut-off values as to when a correction is indicated in post-traumatic rotational deformity of the forearm [11]. In the current study, malrotation of the radius or ulna ≥ 15° was defined as a non-anatomic correction.

Our secondary outcome measures were: functional gain in pro-supination and patient-reported outcome measures (PROMs): the QuickDASH questionnaire (11 items, range 0–100), numerical rating scale (NRS) scores for pain and appearance (range 0–10), and maximal grip strength using a JAMAR hand dynamometer (J.A. Preston Corporation, New York, NY, USA). Pro-supination was measured with a universal goniometer utilizing the method of the American Society of Hand Therapists [12]. Functional outcome was measured by two authors independently (E.E. and J.C.).

### 2.2. 3D Radiographic Assessment

Radiographic evaluation of the accuracy of the performed correction was performed by analyzing the 3D models of the pre- and post-operative forearm bones according to the following steps: using Mimics software (Mimics Research 25.0), segmentation is performed using a threshold-connected region growing algorithm that collects voxels that belong to the affected bone. Then, the forearm bones are extracted as separate 3D objects.

Next, 3-Matic software (3-Matic Research 17.0) was used to compare 3D models of the pre-operative situation, planned correction, and post-operative result. First, analytic cylinders of the proximal and distal shafts of the radius and ulna are created to establish the axis of the proximal and distal parts of both bones in all three situations. Next, using a closest fit algorithm, the proximal ends of the radius and ulna of all three situations are aligned proximally. The axes of the proximal shaft proximal to the planned correction were used for the coordinate system, as this axis was alike in all three situations. Finally, the deviation between the distal segments in all three situations was measured to assess the degree of angular and rotational malalignment in the coronal, sagittal, and axial planes. The coordinate system of the radius was established as described by the International Society of Biomechanics (ISB) 2005 recommendations [13]. The maximum deformity angle (MDA) was calculated by combining the measurements of angular deformity in the coronal and sagittal planes, according to the Pythagorean theorem. MDA was calculated from the coronal and sagittal planes derived from CT instead of plain radiographs to increase the accuracy of the measurement because the reliability of measurements from 2D images is hampered by over-projection [14]. Two authors measured radiographic outcomes independently (K.R. and E.E). Mean values of both assessors are presented.

### 2.3. Statistical Analysis

*p*-values < 0.05 were considered statistically significant. The intraclass correlation coefficient (ICC) was measured to assess the inter-observer reliability of the radiographic measurements. One-way analysis of variance (ANOVA) was performed to study the relationship between an anatomic correction and functional outcomes (gain in pro-supination and PROMs). Subsequently, multi-variate linear regression analysis was performed to investigate the relationship between the accuracy of correction (residual MDA and malrotation of radius and ulna) and gain in pro-supination, both on a continuous scale.

## 3. Results

Between October 2016 and July 2018, 3D corrective osteotomies of both the radius and ulna were performed in fifteen patients due to pediatric malunited both-bone diaphyseal forearm fractures. Patients had a mean age at trauma of 9.6 years, a mean time until 3D corrective osteotomy of 5.9 years, and a mean age at osteotomy of 15.5 years. There was a mean operating time of 138 min (SD 35) for the 3D corrective osteotomies of the radius and ulna. In addition, four out of fifteen patients underwent an additional soft-tissue release. There were three minor complications: ulnar plate removal, delayed union, and transient neuropraxia of the superficial radial nerve. There were three minor complications: ulnar plate removal, delayed union, and transient neuropraxia of the superficial radial nerve.

### 3.1. Primary Outcomes

**Figure 3:**
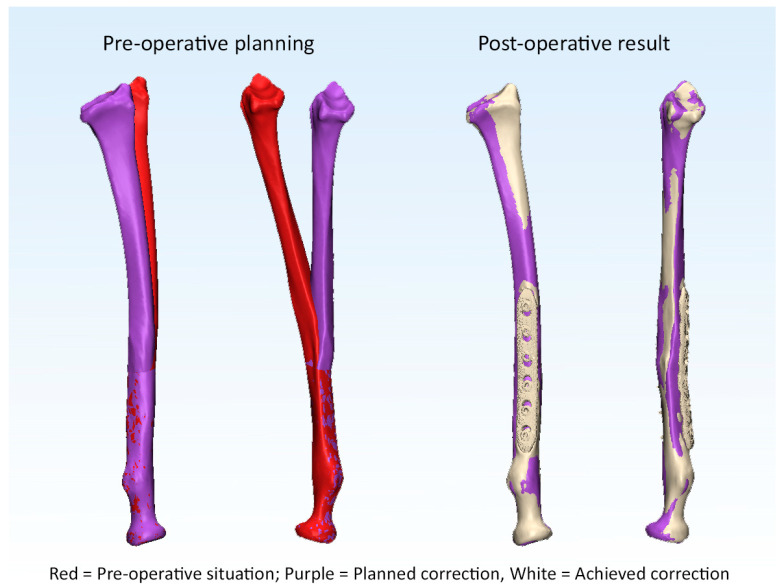
Example of an anatomic correction of the radius.

**Figure 4:**
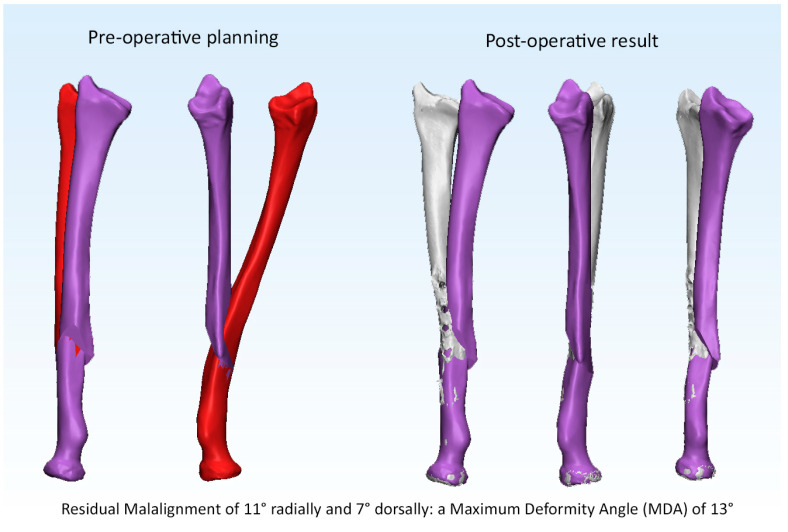
Example of a non-anatomic correction of the radius.

**Figure 5:**
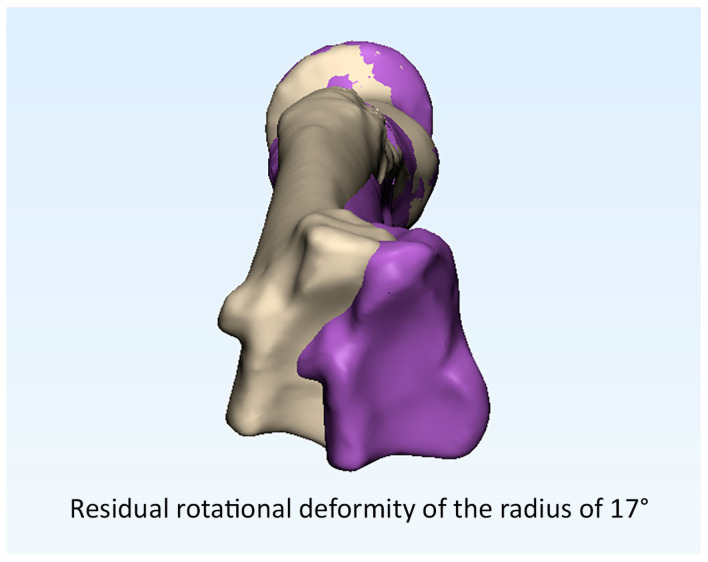
Example of residual malrotation of the radius.

The pre- and post-operative malalignments of the radius and ulna are provided in Table 1. Anatomic correction was achieved in 10 out of 15 patients (25 out of 30 forearm bones) after 3D corrective osteotomy. Examples of an anatomic and a non-anatomic correction of the radius are supplied in Figure 3 and Figure 4 (Case 1 and 4). Likewise, an example of residual malrotation of the radius is provided in Figure 5 (Case 13).

### 3.2. Is Anatomic Correction Associated with Greater Functional Outcomes?

Three dimensional corrective osteotomy provided a mean gain in pro-supination from 67° (44% of contralateral) pre-operatively to 128° (85% of contralateral), thus a mean total gain of 62°. The results of ANOVA are presented in Table 2. ANOVA revealed ten patients who achieved anatomic correction after 3D corrective osteotomies had significantly greater gains in pro-supination than those with non-anatomic corrections: 70° (95% CI: 55–85°) versus 46° (95% CI: 28–64°). Patient-reported outcome measures or grip strength measurements between anatomic and non-anatomic corrections showed no significant differences. Multi-variate linear regression analysis revealed residual malrotation of the radius was associated with inferior pro-supination (*p* = 0.026); the model is provided in Table 3.

In our radiographic assessment, the interrater reproducibility showed intra-class correlations of 0.996 (95% CI: 0.991–0.998) and 0.992 (0.984–0.996) for measurement of the MDA of the radius and ulna; 0.990 (0.979–0.995) and 0.971 (0.938–0.986) for rotational assessment of the radius and ulna.

## 4. Discussion

This prospective study aimed to assess the accuracy of correction after 3D corrective osteotomy for pediatric forearm malunion and if anatomic correction influences the functional outcome. In this study, 3D-planned corrective osteotomies for pediatric malunited both-bone forearm fractures resulted in anatomic corrections in 10 out of 15 patients (25 out of 30 operated forearm bones). Patients with anatomic corrections had statistically significantly greater gains in pro-supination after 3D corrective osteotomies than non-anatomic corrections (70° versus 46°). Residual malrotation of the radius after 3D corrective osteotomy was associated with an inferior gain in forearm rotation.

Understanding the complex 3D deformities of both forearm bones in a malunited forearm fracture remains challenging. Therefore, a 3D corrective osteotomy is a promising technique. Recurrent patterns in forearm malunion are often seen. The supinator, pronator teres, and pronator quadratus muscles exert a pulling force upon fracture fragments, which can lead to angular deformity, malrotation, or narrowing of the interosseous space. In fractures located proximal to the pronator teres insertion, the proximal fragment supinates and flexes due to unopposed forces of the supinator and biceps brachii, whereas the distal fragment pronates due to the pronator quadratus and pronator teres. In contrast, in fractures located distal to the pronator teres insertion, the proximal fragment will not rotate as the supinator opposes the forces of the pronator teres and biceps brachii. The distal fragment will pronate and deviate towards the ulna due to the pronator quadratus [4]. Angular deformities of the radius and ulna lead to bony impingement or increased interosseous membrane (IOM) tension, which causes impairment in forearm rotation [15]. In a cadaveric study, a dorsal angular deformity of 20° caused a limitation in pronation. Correspondingly, a volar angular deformity of 20° led to supination limitation. Lastly, angular deformity narrowing the interosseous space limited both pro- and supination [16]. In 2018, Abe et al. stated a pronation limitation was found if there was bony impingement due to dorsal angulation of the radius (>8°) because the interosseous space is encroached during pronation [17]. A supination limitation was found if there was a tightness of the transverse central band (CB) due to valgus deformity of the ulna (>6°), which increases the interosseous space during supination.

Unfortunately, there is no published literature with CT-based accuracy assessment of conventional 2D planned corrective osteotomies with which to compare.

In 2008, Murase and colleagues reported the accuracy of 3D corrective osteotomy for malunited forearm fractures in 10 patients. The mean angle of deformity improved from 16° pre-operatively to 1° after surgery. The mean pro-supination improved from 79° to 155° post-operatively.

In 2012, Miyake et al. published the outcomes of 3D corrective osteotomies for malunited forearm fractures in 20 patients. The average radiographic deformity improved from 21° pre-operatively to 1° post-operatively. In addition, their forearm motion improved from 76° pre-operatively to 152° post-operatively.

In 2013, Kataoka et al. published the results of 3D corrective osteotomies with PSIs for malunited forearm fractures in four patients. They used standard plates, which were pre-bent to fit around 3D-printed, real-sized plastic bone models of the radius and ulna. They achieved an accuracy of correction with a mean error in all directions of <2° for both the radius and the ulna. Mean errors were greater in growing children, as longitudinal forearm growth was not considered. They achieved a mean gain in pro-supination from 106° pre-operatively to 158° post-operatively [18].

In 2015, Bauer et al. performed 19 3D corrective osteotomies due to forearm deformity in children of which 15 were post-traumatic. In their study, maximum deformity angulation of the radius and ulna improved from 23° and 23° to 9° and 8°, respectively. Ten patients were operated on due to limited pro-supination, and a gain in pro-supination was seen from 85° to 138°.

In 2017, Byrne et al. published the outcomes of five patients who underwent 3D corrective osteotomies for malunited diaphyseal forearm fractures. Besides 3D-printed PSIs, they also used patient-specific plates. They found a mean error in the correction of 1.4° for the radius and 1.8° for the ulna. They aimed to correct angular deformities within 5° of the contralateral side and succeeded in 80% of cases. In addition, 3D corrective osteotomy improved mean pro-supination from 115° to 176°.

In 2019, Oka et al. performed 16 3D corrective osteotomies for malunited fractures of the upper extremity. They also used patient-matched plates. They achieved a correction to within 5° of contralateral in 15 of 16 patients after 3D corrective osteotomies. In their study, the mean difference between the planned correction and the achieved result was <1° in all three planes. In patients who were operated on due to limited pro-supination, a gain in pro-supination was seen from 115° to 162°.

In our series, the 3D osteotomy to correct a pediatric forearm malunion provided a highly accurate correction comparable to the studies mentioned above. Anatomic corrections were associated with greater gains in pro-supination. Thus, a lesser gain in forearm rotation was seen if a greater residual angular or rotational deformity persisted after 3D corrective osteotomy. Besides the highly accurate correction and excellent functional outcomes, another potential advantage of 3D modeling and 3D printing is to improve the patient–doctor relationship by giving them insights into the deformity’s complexity and the surgical procedure’s goal [19].

In our study, residual malrotation of the radius was associated with inferior pro-supination. Not restoring the natural radial bow may lead to bony impingement or too tight soft-tissue, which hinders the radius from swiveling around the ulna. In 1984, Tarr et al. claimed any torsional deformity of the radius leads to a loss of forearm rotation equal to the magnitude of the rotational malalignment but in the opposite direction [16]. However, in a cadaveric study by Kasten et al., a rotational malalignment of the radius of 30° in pronation resulted in a supination deficit of only 14°. Similarly, a rotational malalignment of 30° in supination resulted in a pronation deficit of only 11° [20]. Malrotation of the ulna is well tolerated since the ulna is a relatively straight bone. Thus, this leads to less restriction in forearm rotation than malrotation of the radius [11,21]. A study by Tynan et al. created malrotations of the ulna of 30°, which led to a decrease in forearm rotation of less than 20° [21].

In our study, there were a few cases with considerable residual malalignment or malrotation (Cases 1, 2, 3, 4, and 13). Although all patients were operated on by two experienced orthopedic hand surgeons operating together, four out of five non-anatomic corrections occurred in the first four operated patients. This suggests a considerable learning curve exists for 3D corrective osteotomy for diaphyseal both-bone forearm malunion. Therefore, a larger series is needed to detect if the surgical experience is a source of bias in the accuracy of a 3D corrective osteotomy. Oka et al. stated, “The simple surgical procedure is another advantage of the use of PMIs” [3]. However, we advocate there are still many possible challenges during surgery. For example, the absence of bony landmarks on the forearm bones and additional soft-tissue hindrance may impede the optimal guide position, which may result in under- or over-correction, as suggested by Jeuken et al. [22].

We did not expect residual malalignment or malrotation. The drilling guides dictate screw placement proximal and distal of the planned osteotomy. They are designed with the correct amount of rotational and angular correction built in so once the osteotomies are completed, the placement of screws should provide the desired correction [23].

Therefore, we investigated our outliers in more detail. There were no manufacturing issues. Three out of five non-anatomic corrections were malunions in the proximal diaphysis, suggesting a relation with a more complex surgical approach and more soft tissue hindering snug fit positioning of the surgical guides. Furthermore, the pre-operative plan for 3D corrective osteotomy does not consider the soft-tissue issues seen in post-traumatic forearm malunion. If there is a long interval between trauma and osteotomy in a growing child, soft-tissue contractures of the IOM, proximal and distal radioulnar joint capsule (DRUJ) can be seen [5]. Previously, persisting deficits in pro-supination after corrective osteotomy in longstanding forearm malunions have been seen, regardless of full geometric restoration of bony anatomy [2,24]. The IPD meta-analysis results supported soft tissue contracture’s role in a longstanding malunion [25]. A long interval between trauma and corrective osteotomy compromised the functional gain in pro-supination, which was confirmed in our previous publication [8].

### Limitations

This study has some limitations. First, there was no control group that underwent conventional corrective osteotomy using 2D radiographic planning without patient-specific 3D printed surgical guides. However, we find using only 2D radiographic planning for the correction of a 3D deformity unethical, as inferior results can unequivocally be expected. A previous meta-analysis showed the use of 3D computer-assisted techniques is a predictor of superior functional outcome after corrective osteotomy for a malunited pediatric forearm fracture [25].

Additionally, we included a relatively small number of patients. However, severe limitation in forearm rotation due to a pediatric malunited both-bone forearm fracture fortunately occurs seldomly. Therefore, a corrective osteotomy is rarely indicated.

Another limitation is if 3D corrective osteotomy did not provide full pro-supination, additional IOM or DRUJ release was performed during surgery. Thus, post-operative outcomes were not solely determined by correcting the bony anatomy. In the previous studies, no additional soft-tissue releases were performed [2,3,5,6,18,23]. Yet, this surgical plan does reflect our clinical approach to treating a post-traumatic forearm rotation: correct the bony deformity first, then solve the soft-tissue problems.

Furthermore, the post-operative CT scan was obtained one year after surgery. Thus, in children with remaining growing potential, additional remodeling could occur. Eight out of fifteen patients were aged <15 years at the time of 3D corrective osteotomy.

Lastly, there were only a few outliers to investigate due to the overall high accuracy of the correction and excellent functional outcome after 3D corrective osteotomy. Therefore, perhaps there are other unknown predictors for an inferior outcome we have yet to identify. Larger series are needed.

## 5. Conclusions

Three-dimensional corrective osteotomy using patient-specific instruments results in an accurate correction of pediatric malunited forearm fractures. A close to normal pro-supination was obtained in the majority of patients. Patients with an anatomic correction of the radius had better forearm rotation than non-anatomic corrections. Residual malrotation of the radius after a 3D corrective osteotomy is associated with an inferior outcome. Although PSIs simplify the operative procedure, a considerable learning curve still exists for 3D corrective osteotomy.

Desirable future research is a randomized controlled trial (RCT) comparing the outcomes after 3D-planned corrective osteotomy with or without PSIs because cost increases are substantially due to the 3D printing of PSIs. Future studies on 3D corrective osteotomy should provide patient-reported outcomes measures, functional outcomes, as well as radiographic outcomes on the accuracy of the achieved correction.

## Figures and Tables

**Table 1:** Radiographic outcomes: pre- and post-operative malalignment (°).

	Radius								Ulna							
	Pre-Operative				Final Follow-Up				Pre-Operative				Final Follow-Up			
Pt	Cor	Sag	MDA	Ax	Cor	Sag	MDA	RM	Cor	Sag	MDA	Ax	Cor	Sag	MDA	RM
1	3	14	14	−6	0	0	0	6	6	−5	7	32	−5	5	7	−12
2	9	22	23	24	0	0	1	−2	16	1	16	−1	1	0	1	15
3	−4	8	8	−11	−3	0	3	−9	8	−6	11	−4	1	0	1	−16
4	0	17	17	−26	11	7	13	−6	8	−20	21	11	1	−1	2	2
5	−2	22	23	−8	−1	0	1	14	9	−7	11	0	0	0	0	4
6	7	27	27	−31	0	−3	3	−3	8	−18	19	6	1	0	1	3
7	−11	0	11	−13	1	−1	1	−4	−7	19	20	3	0	1	1	−5
8	5	18	19	18	−1	−1	1	−8	11	2	11	−1	−1	0	1	3
9	0	16	16	1	−1	−1	2	10	6	−5	8	−7	0	−1	1	0
10	17	24	29	−4	0	−2	2	−5	1	−13	13	13	−1	−1	2	9
11	6	19	20	−12	3	3	5	0	3	−15	15	−4	0	0	0	0
12	1	11	11	49	−1	0	1	−1	5	−3	6	−3	−1	−4	4	5
13	9	4	10	15	−2	4	5	17	7	2	7	−20	0	2	2	0
14	−2	6	6	−5	1	1	1	−10	13	−1	13	−7	0	1	1	−1
15	−7	3	7	−17	2	0	2	−3	4	3	5	5	−2	−1	3	−6
**Mean**	**8** **.** **1**	**14.0**	**16.1**	**15.9**	**1.8**	**1.7**	**2.6**	**6.6**	**7.4**	**8.0**	**12.2**	**7.7**	**1.0**	**1.2**	**1.7**	**5.5**
**SD**	**9.4**	**8.4**	**7.2**	**12.4**	**2.7**	**2.0**	**3.1**	**4.8**	**3.7**	**6.9**	**5.2**	**8.7**	**1.2**	**1.4**	**1.7**	**5.3**

Cor = coronal plane; Sag = sagittal plane; Ax = axial plane. MDA = maximum deformity angle; RM = residual malrotation. Dorsal angulation = positive; volar = negative; radial = positive; ulnar = negative; axial malrotation in pronation = positive; axial malrotation in supination = negative. Means are calculated based on absolute values.

**Table 2:** ANOVA.

	Anatomic Correction (*n* = 10)	Non-Anatomic Correction (*n* = 5)	*p* =
Pre-op pro-supination	67° (53–80°)	66° (36–97°)	0.97
Pro-supination at FU	136° (125–148°)	112° (95–129°)	0.01
Gain in pro-supination	70° (55–85°)	46° (28–85°)	0.04
Pre-op QUICKDASH	22 (13–30)	31 (19–43)	0.16
QUICKDASH at FU	13 (10–16)	17 (14–20)	0.07
∆ QUICKDASH	8 (0–17)	14 (2–26)	0.38
NRS pain score	1.1 (−0.5–2.7)	3.0 (−0.4–6.4)	0.18
NRS cosmetics	2.3 (0.5–4.2)	4.6 (1.3–8.0)	0.13
Grip strength (%)	94 (88–98)	90 (78–102)	0.41

Confidence interval of 95% presented as: (95% CI); FU: follow-up; NRS: numeric rating scale.

**Table 3:** Multi-variate linear regression.

	Unstandardized Coefficients		
Model	B	Std. Error	Significance
(Constant)	79.0	8.4	<0.001
Residual malrotation Radius	−2.6	1.1	0.026

Dependent variable: gain in pro-supination.

## Data Availability

Data are contained within the article.
